# Homogeneous Canine Chest Phantom Construction: A Tool for Image Quality Optimization

**DOI:** 10.1371/journal.pone.0154193

**Published:** 2016-04-21

**Authors:** Ana Luiza Menegatti Pavan, Maria Eugênia Dela Rosa, Guilherme Giacomini, Fernando Antonio Bacchim Neto, Seizo Yamashita, Luiz Carlos Vulcano, Sergio Barbosa Duarte, José Ricardo de Arruda Miranda, Diana Rodrigues de Pina

**Affiliations:** 1 Department of Physics and Biophysics, Biosciences Institute of Botucatu, São Paulo State University, Botucatu, São Paulo, Brazil; 2 Department of Tropical Diseases and Diagnostic Imaging, Botucatu Medical School, São Paulo State University, Botucatu, São Paulo, Brazil; 3 Department of Animal Reproduction and Veterinary Radiology, School of Veterinary Medicine and Animal Science, São Paulo State University, Botucatu, São Paulo, Brazil; 4 Brasilian Center of Physics Research, Rio de Janeiro, Rio de Janeiro, Brazil; Université de Poitiers, FRANCE

## Abstract

Digital radiographic imaging is increasing in veterinary practice. The use of radiation demands responsibility to maintain high image quality. Low doses are necessary because workers are requested to restrain the animal. Optimizing digital systems is necessary to avoid unnecessary exposure, causing the phenomenon known as *dose creep*. Homogeneous phantoms are widely used to optimize image quality and dose. We developed an automatic computational methodology to classify and quantify tissues (i.e., lung tissue, adipose tissue, muscle tissue, and bone) in canine chest computed tomography exams. The thickness of each tissue was converted to simulator materials (i.e., Lucite, aluminum, and air). Dogs were separated into groups of 20 animals each according to weight. Mean weights were 6.5 ± 2.0 kg, 15.0 ± 5.0 kg, 32.0 ± 5.5 kg, and 50.0 ± 12.0 kg, for the small, medium, large, and giant groups, respectively. The one-way analysis of variance revealed significant differences in all simulator material thicknesses (*p* < 0.05) quantified between groups. As a result, four phantoms were constructed for dorsoventral and lateral views. In conclusion, the present methodology allows the development of phantoms of the canine chest and possibly other body regions and/or animals. The proposed phantom is a practical tool that may be employed in future work to optimize veterinary X-ray procedures.

## Introduction

The use of radiation in veterinary medicine demands responsibility to maintain rigid radiation safety standards and practice. Workers are often requested to restrain animals during the procedure to avoid movement artifacts and maintain image quality [1, 2]. Therefore, the exposure of workers have to be minimized considering the radiation protection principles because of their presence in the examination room throughout the procedure [3–5]. In such situations, scattered radiation represents the main source of radiation that is received by operators [1].

With the increasing use of digital radiographic imaging in medical and veterinary practice, a phenomenon known as *dose creep* has been reported [6, 7]. Dose creep is the gradual increase exposure factors and dose over time for a given radiographic anatomical projection. The phenomenon occurs due to the ability of the wider dynamic range of the system and computer processing of the acquired image to correct inappropriate exposure parameters [7–9].Therefore, optimize digital systems to maximize image quality and minimize personnel exposure is important [3].

Optimization procedures have been widely exploited for humans in the literature [1, 10–14] Homogeneous phantoms are the simplest tool that is used for optimization purposes [15, 16]. Homogeneous phantoms are extensively employed to assess objective metrics associated with image quality, such as the signal difference-to-noise ratio (SdNR) [12, 14]. A higher SdNR provides superior image quality compared with a lower SdNR [12, 14].

These phantoms are constructed of tissue-equivalent materials with tridimensional structures to simulate the absorption and scattering of X-ray beams in the body. Homogeneous phantoms are generally constructed of polymethyl methacrylate (Lucite) and aluminum through the classification and quantification of tissues from computed tomography (CT) exams [15].

However, the development of homogeneous phantoms for veterinary patients has remained unexplored. Therefore, the aim of this work was develop homogeneous canine thorax phantoms for the dorsoventral and lateral views for different sizes of dogs. These phantoms may be applied for optimization processes and to improve image quality, reduce personnel exposure minimizing the occurrence of dose creep.

## Materials and Methods

### Database

The present study was developed with approval from the Ethics Committee on the Use of Animals of the authors’ institutions and national review panels under protocol no. 90/2014. The database consisted of 356 retrospective chest CT exams of dogs, which were veterinary patients from the Diagnostic Imaging Section of the Veterinary Hospital, University of Veterinary Medicine and Animal Science (HV/FMVZ-Unesp Botucatu), between January 2007 and May 2015. Veterinary patients with significant thoracic disease, including primary pulmonary masses, pneumothorax, or pleural effusion, were excluded. Veterinary patients with superimposed nodules or a high number of pulmonary nodules to be counted reliably were also excluded. Therefore, 276 patients were excluded as described, leaving 80 for following analyses.

The veterinary patients were separated into groups of 20 dogs each according to weight: small (S) group (≤ 9.0 kg), medium (M) group (9.0–23.0 kg), large (L) group (23.1–40.0 kg), and giant (G) group (> 40.1 kg).[17] Mean weights were 6.5 ± 2.0 kg, 15.0 ± 5.0 kg, 32.0 ± 5.5 kg, and 50.0 ± 12.0 kg, for the S, M, L, and G groups, respectively.

### Data acquisition

The CT scans were acquired using a Shimadzu SCT-78000 Helical scanner (Shimadzu, Kyoto, Japan) with the following parameters: 0.59 × 0.59 mm to 0.80 × 0.80 mm pixel size, 512 × 512 pixel matrix, 5.0 mm interval between slices, 3.0–7.0 mm slice width, and 120 kV tube voltage.

### Automatic computational algorithm

We used an established methodology [10, 15, 18] to develop an automatic computational algorithm that is able to classify and quantify biological tissues of the canine chest based on retrospective CT exams. The slices from CT exam were used to obtain the amount of each biological tissue present in the studied region, which was converted in simulator materials to construct the homogeneous phantoms. For each canine group, we developed two types of phantoms (dorsoventral chest phantom and lateral chest phantom) to simulate two common projections that are used in clinical practice. The construction of the phantoms is described below.

#### Histogram of CT exams

**Fig 1:**
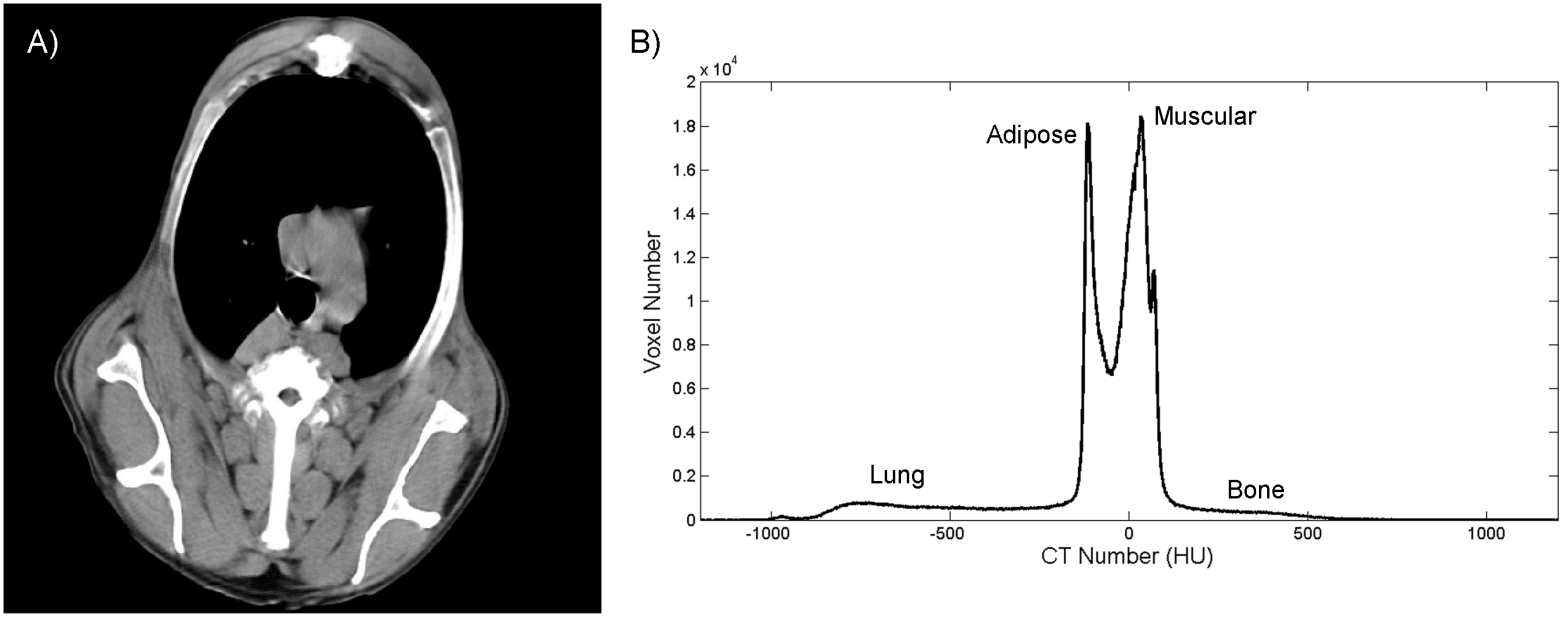
(i) Slice from canine chest CT exam. (ii) CT number distribution histogram.

Each CT slide was automatically aligned to correct possible dog positioning errors. Based on differences in the attenuation of each tissue in the CT slice (Fig 1A), represented by CT number (Hounsfield Units [HU]), the algorithm classified each voxel as belonging to a given tissue. This classification was based on the histogram of CT number. Fig 1B illustrates the histogram of image shown in Fig 1A that presents four well-separated characteristic peaks of different tissues: lung tissue, adipose tissue, muscle tissue, and bone.

#### Classification and quantification of chest tissues by Gaussian curves

**Fig 2:**
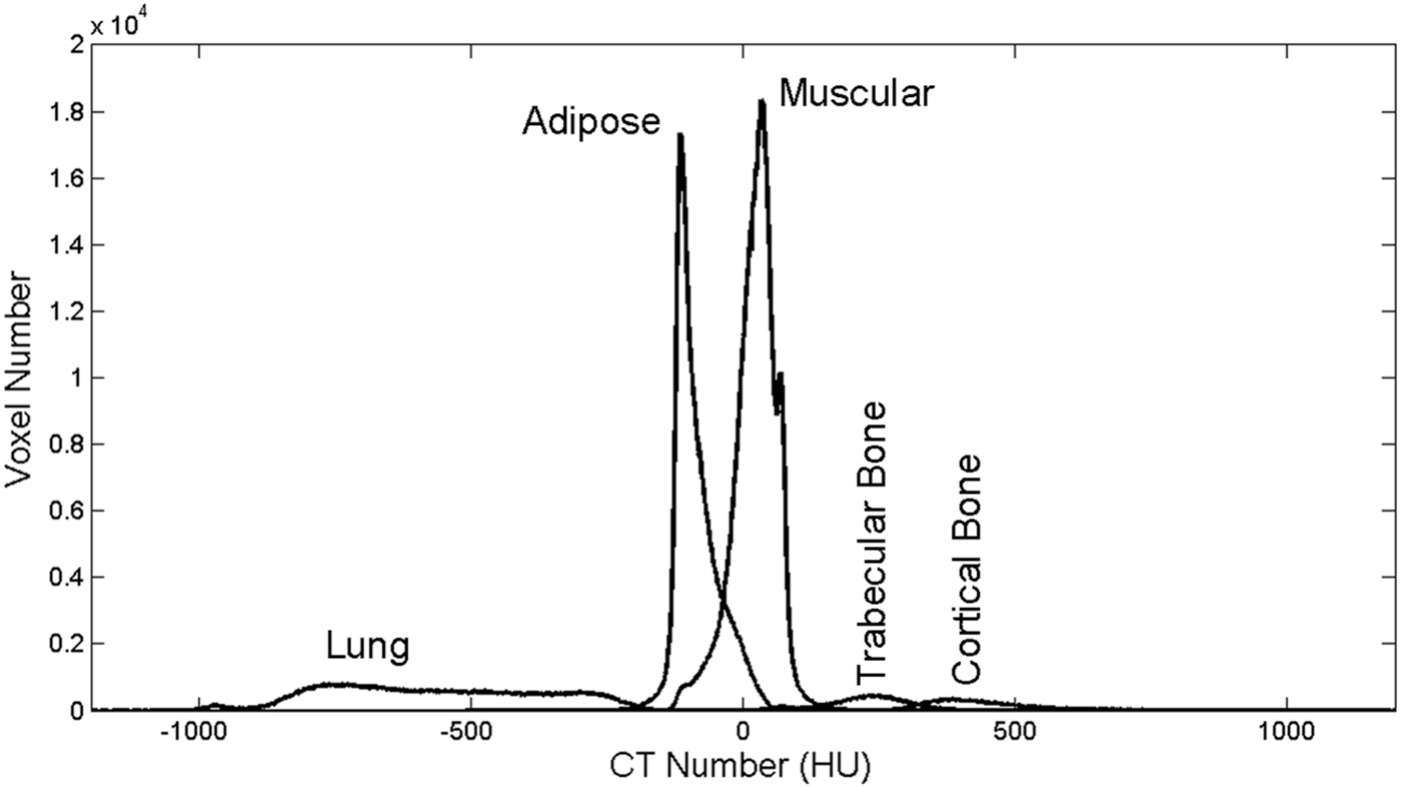
Histogram divided by Gaussian distribution curves to classify tissues as lung tissue, adipose tissue, muscle tissue, and trabecular/cortical bone.

To automatically classify each voxel from the histogram, the algorithm used Gaussian distribution curves as the base. These curves represent the degree of accuracy of a voxel that belongs to a given tissue (adipose tissue, muscle tissue, lung tissue, and bone) according to CT number. An experienced veterinary radiologist manually measured the mean and standard deviation of the CT number for each tissue. The measured values were used to create Gaussian distribution curves. Fig 2 shows the histogram of Fig 1B divided by the curves that were created: -600 HU indicates lung tissue, -100 HU indicates adipose tissue, 100 HU indicates muscle tissue, and > 300 HU indicates bone. Trabecular and cortical bones presented significant differences in X-ray attenuation and thus were divided into two Gaussian curves with peaks at 300 HU and 400 HU, respectively. Therefore, each voxel that belonged to a histogram was classified as a specific tissue according to the Gaussian distribution curve.

To estimate the amount of tissue (*i*) in the exam, the number of voxels (N_v_) that belonged to specific tissues (*i)* was used. N_v_(*i*) was calculated by multiplying the examination histogram by the corresponding Gaussian distribution curve. N_v_(*i*) was used to obtain the mean tissue thickness according to the following equation:

$$T\left(i\right)=\frac{N_{v}\left(i\right)A_{p}}{S\;D}\;,$$ (1)

where T(*i*) is the mean thickness of tissue (mm), *i* is the tissue type (lung, adipose, muscle, or bone), A_p_ is the pixel area (mm^2^), S is the number of slices, and D is the mean diameter of the chest CT exam (mm).

The multiplication of N_v_ with A_p_ results in the total tissue (i) area (mm^2^). The mean tissue area (mm^2^) for each slice results from the total tissue area divided by S. To obtain the thickness of tissue (T(*i*)) in the X-ray beam direction, the mean area is divided by the mean perpendicular diameter, *i*.*e*. to develop a dorsoventral type phantom, the perpendicular diameter used in the equation was the mean lateral diameter. Likewise, to develop a lateral type phantom, the dorsoventral diameter was employed.

Finally, the mean thicknesses of the biological tissues were converted into the corresponding thicknesses of the simulator material plates. Lungs were simulated by air. Soft tissue (muscle tissue and adipose tissue) was simulated by Lucite. Bone (trabecular and cortical) was simulated by aluminum. This process was based on literature [19] using quantification values [20] for the composition of biological tissues.

### Development of homogeneous canine chest phantoms

**Fig 3:**
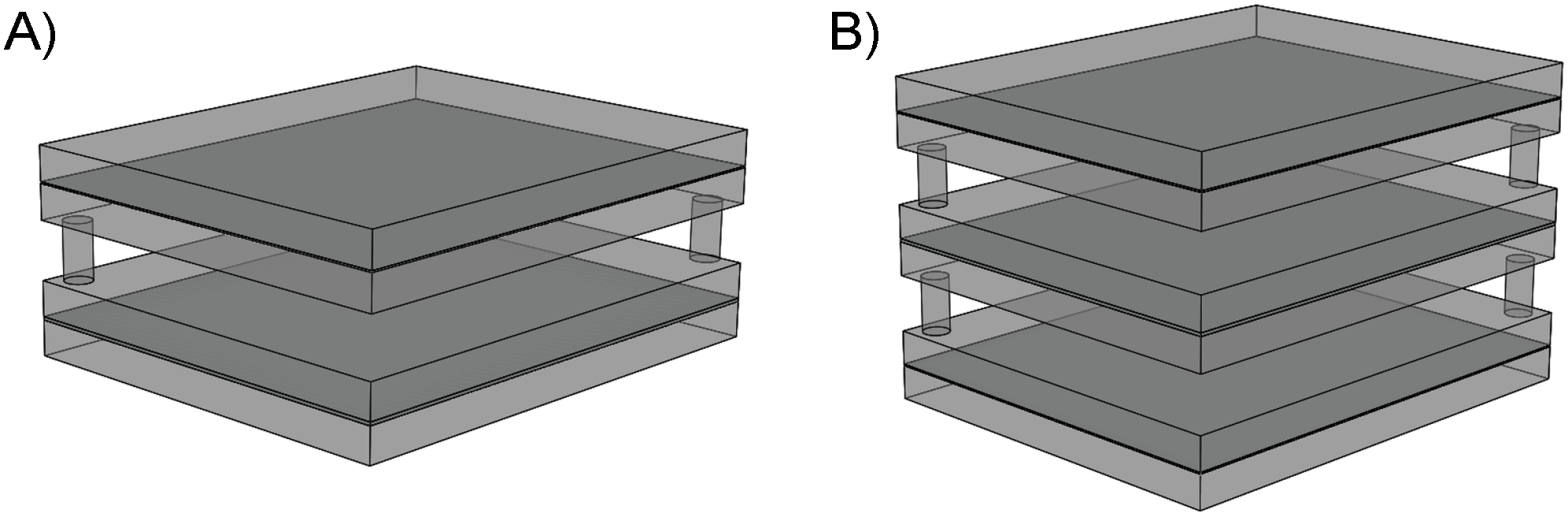
Homogeneous canine chest phantom configuration for (i) dorsoventral and (ii) lateral views. The phantoms consisted of Lucite slabs that simulated soft tissues (muscle tissue and adipose tissue) and aluminum foil between the Lucite slabs that simulated bone. An air gap was inserted to simulate lung tissue.

The dorsoventral and lateral homogeneous canine chest phantoms were constructed using the achieved thicknesses of simulator material and are shown in Fig 3. The dorsoventral homogeneous canine chest phantom (Fig 3A) was constructed of aluminum and Lucite plates. The first Lucite pair (on the top) sandwiched an aluminum plate. The Lucite thickness was distributed into four equal slabs. The second Lucite pair (on the bottom) contained a slightly wider aluminum slab inside. Between the upper and lower pairs of plates, spacers were inserted that represented lung tissue as an air gap. The architecture of the canine chest phantom is similar to an equivalent patient phantom [16, 21] that is used for optimization purposes in human exams.

The architecture of the lateral homogeneous canine chest phantom was based on literature [22]. As shown in Fig 3B, the phantom was separated into three parts. Each part had an aluminum plate sandwiched by two Lucite plates. The aluminum that was placed in the intermediate pair accounted for the amount of bone from the vertebral column and extern. Air gaps were inserted between the pairs.

The dimensions (length and width) of the phantoms were established as the mean dorsoventral and lateral-lateral canine size. The thicknesses and dimensions of the plates were separately calculated for each canine group.

### Statistical analysis

The thicknesses of each simulator material was compared between different dog groups to assess whether the phantoms follow the same division of canine groups based on weight.

The statistical analysis of the Gaussian distribution data was performed using one-way repeated-measures analysis of variance (ANOVA) followed by Student’s *t*-test for comparisons between canine groups for each simulator material. Values of *p* < 0.05 were considered statistically significant. This statistical analysis was performed for both phantom types (dorsoventral and lateral). The results are presented as mean and standard deviation.

## Results

Tables 1 and 2 show the thicknesses for both phantoms (dorsoventral and lateral) according to canine group. Each canine group was characterized by the average thickness of biological tissue (Ti), thickness of the simulator material (TS), coefficient of variation (defined as the standard deviation of the obtained examination thickness divided by the mean value), and mean dimensions (length x width) of the phantom.

**Table 1:** Mean Thickness of Biological Tissue (Ti) in the Examination, Thickness of Simulator Material (TS), Coefficient of Variation, and Mean Dorsoventral Phantom Dimensions (length x width).

Group weight (kg)	Tissue	Ti (mm)	Simulator Material	TS (mm)	Coefficient of Variation	Mean Phantom Size (cm^2^)
**Small (< 9)**	Lung	20.61	Air	20.61	0.30	15 × 15
	Total Soft	50.26	Lucite	63.25	0.22	
	Total Bone	8.93	Aluminum	1.55	0.25	
**Medium (9–23)**	Lung	28.61	Air	28.61	0.21	20 × 20
	Total Soft	77.36	Lucite	97.34	0.19	
	Total Bone	14.47	Aluminum	2.47	0.21	
**Large (23–40)**	Lung	34.14	Air	34.14	0.19	25 × 25
	Total Soft	98.43	Lucite	123.86	0.11	
	Total Bone	17.19	Aluminum	2.99	0.21	
**Giant (> 40)**	Lung	36.52	Air	36.52	0.13	30 × 30
	Total Soft	123.86	Lucite	155.86	0.14	
	Total Bone	20.05	Aluminum	3.49	0.10	

**Table 2:** Mean Thickness of Biological Tissue (Ti) in the Examination, Thickness of Simulator Material (TS), Coefficient of Variation, and Mean Lateral Phantom Dimensions (length x width).

Group weight (kg)	Tissue	Ti (mm)	Simulator Material	TS (mm)	Coefficient of Variation	Mean Phantom Size (cm^2^)
**Small (< 9)**	Lung	24.23	Air	24.23	0.28	13 × 13
	Total Soft	59.20	Lucite	74.49	0.24	
	Total Bone	10.66	Aluminum	1.86	0.30	
**Medium (9–23)**	Lung	30.42	Air	30.42	0.26	18 × 18
	Total Soft	82.48	Lucite	103.78	0.24	
	Total Bone	14.63	Aluminum	2.54	0.24	
**Large (23–40)**	Lung	37.19	Air	37.19	0.21	22 × 22
	Total Soft	109.66	Lucite	137.99	0.27	
	Total Bone	18.57	Aluminum	3.23	0.22	
**Giant (> 40)**	Lung	39.79	Air	39.79	0.22	35 × 25
	Total Soft	134.54	Lucite	169.29	0.21	
	Total Bone	21.79	Aluminum	3.79	0.20	

**Fig 4:**
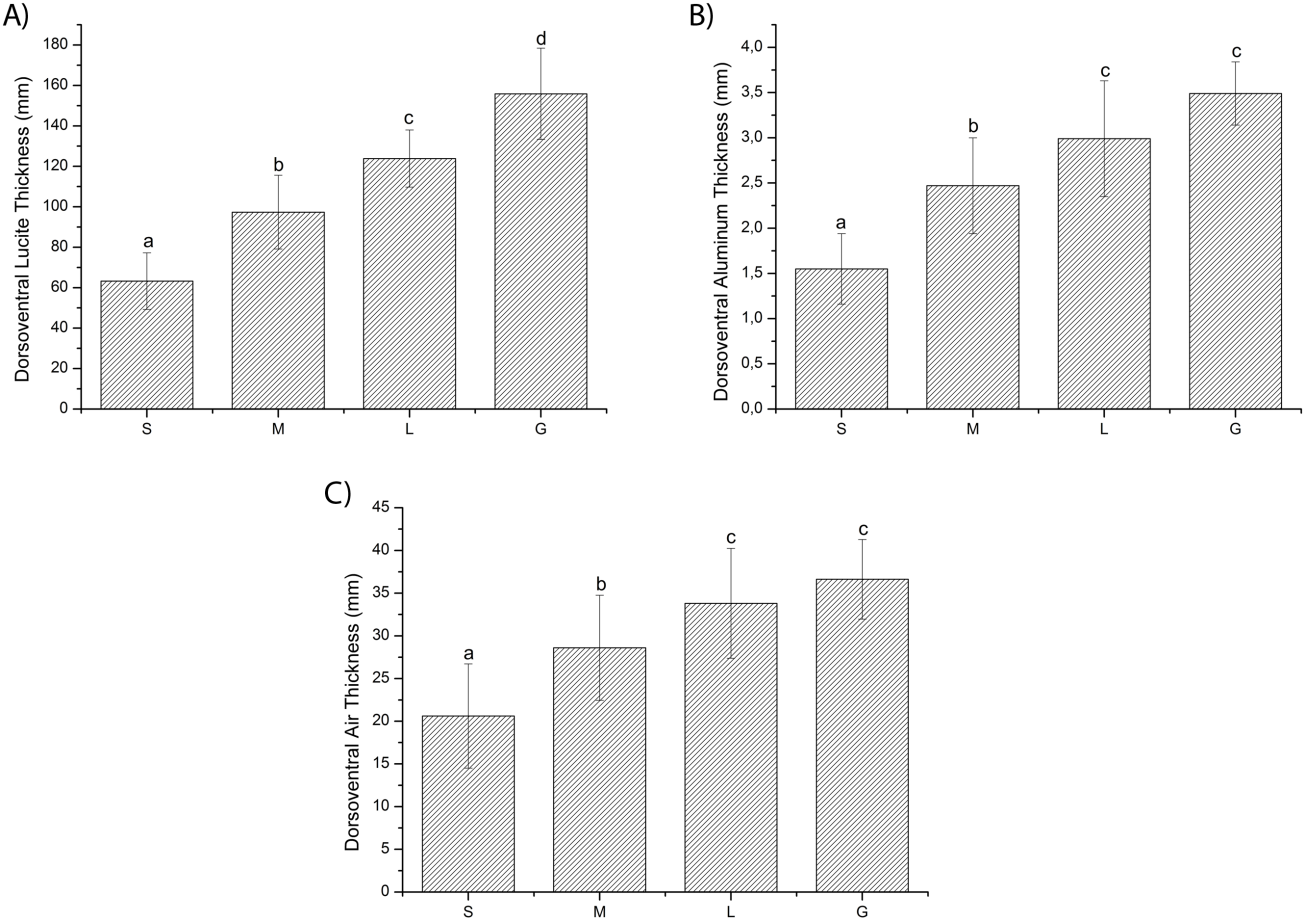
Dorsoventral (i) Lucite, (ii) aluminum, and (iii) air thicknesses for different canine groups. S, small; M, medium; L, large; G, giant. Bar plot of means for each level of the independent variable of a one-way analysis of variance (ANOVA). The data are expressed as mean ± standard deviation. Bars sharing the same letter are not significantly different according to Student’s *t*-test. Differences were considered significant for *p* < 0.05.

**Fig 5:**
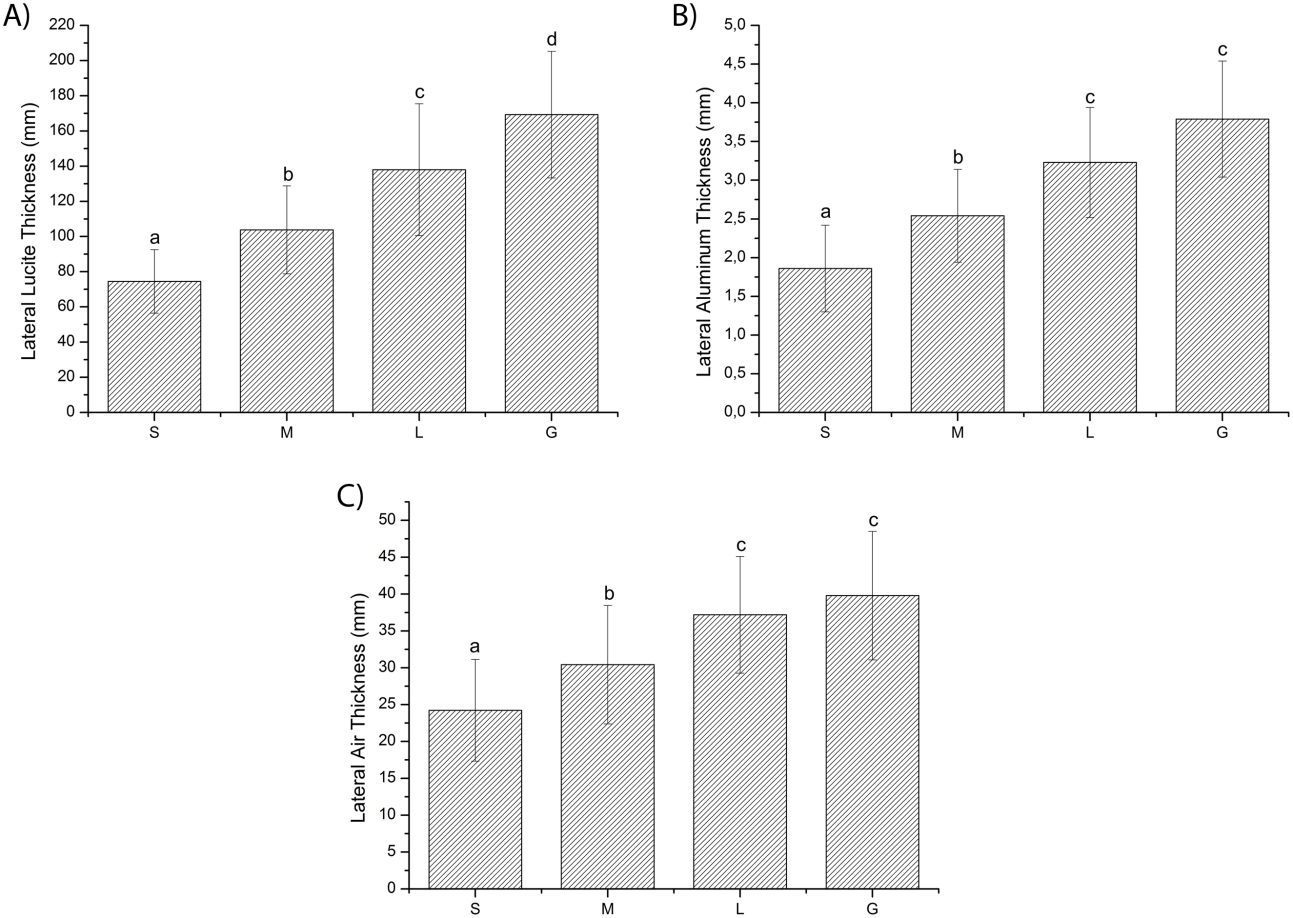
Lateral (i) Lucite, (ii) aluminum, and (iii) air thicknesses for different canine groups. S, small; M, medium; L, large; G, giant. Bar plot of means for each level of the independent variable of a one-way analysis of variance (ANOVA). The data are expressed as mean ± standard deviation. Bars sharing the same letter are not significantly different according to Student’s *t*-test. Differences were considered significant for *p* < 0.05.

For both phantom types (dorsoventral and lateral), the one-way ANOVA revealed significant differences in Lucite, aluminum, and air thicknesses between groups (*p* < 0.05). Student’s *t*-test revealed significant differences between groups (*p* < 0.05) for all materials, with the exception of aluminum and air between the L and G groups (Figs 4 and 5).

## Discussion and Conclusion

Optimizing digital radiographic technique settings is important for animal imaging to maximize image quality while minimizing personnel radiation exposure [3] according to As Low As Reasonably Achievable (ALARA) principles.

In the present study, a robust algorithm widely used in the literature [10, 15, 18] was adapted for veterinary patients and implemented to classify and quantify the amount of tissue in the canine chest. As a result of the quantification, dorsoventral and lateral homogeneous canine phantoms were constructed according to canine group. Tables 1 and 2 show significant coefficient of variation. Such variations within the same canine group may be attributable to differences in nutritional status, breed, and age. Furthermore, deviations in the mean air thickness can result from changes in lung volume during inspiration and expiration.

The statistical analysis for both phantom views revealed significant differences between canine groups for all simulator materials. Student’s *t*-test revealed significant differences for all of the Lucite analyses. For aluminum and air, all of the differences were significant, with the exception of the L and G groups. Such results may be attributable to the same factors that caused significant coefficient of variation. Differences revealed by statistical analysis and variations in the dimensions of the phantoms (length and width) were the main reasons for constructing four different phantoms for each phantom view.

Many studies have been performed to optimize X-ray exams using homogeneous phantoms for humans [10, 12, 14, 23]. However, optimization is still neglected in veterinary medicine. Constructing a homogeneous canine phantom is important to adhere to ALARA principles and minimize dose creep. These phantoms may be used to optimize image quality and dose using such metrics as the signal difference-to-noise ratio, figure of merit [12, 14], modulation transfer function, and effective detective quantum efficiency [10, 24].

In conclusion, we developed eight phantoms of the canine chest using established methodology. Although we used a canine chest model, this methodology may be implemented for other anatomical structures and/or animals. Each of these phantoms were constructed of readily available materials [10].

The homogeneous canine chest phantom that was developed herein is a practical tool that may be employed in future work to optimize veterinary X-ray procedures and avoid the dose creep phenomenon.

## References

[1] Barber J, McNulty JP. Investigation into scatter radiation dose levels received by a restrainer in small animal radiography. Journal of Small Animal Practice. 2012;53:578–85. 10.1111/j.1748-5827.2012.01257.x 22861077

[2] Veneziani GR, Matsushima LC, Fernandez RM, Rodrigues LL. Thermoluminescence measurements of entrance surface skin dose in exams of dog's chest in veterinary radiology. Radiat Meas. 2010;45(3–6):733–5. 10.1016/j.radmeas.2010.02.020 WOS:000279183200139.

[3] Copple C, Robertson ID, Thrall DE, Samei E. Evaluation of two objective methods to optimize kVp and personnel exposure using a digital indirect flat panel detector and simulated veterinary patients. Vet Radiol Ultrasound. 2013;54(1):9–16. 10.1111/j.1740-8261.2012.01989.x . 23293957

[4] Hernandez-Ruiz L, Jimenez-Flores Y, Rivera-Montalvo T, Arias-Cisneros L, Mendez-Aguilar RE, Uribe-Izquierdo P. Thermoluminescent dosimetry in veterinary diagnostic radiology. Appl Radiat Isot. 2012;71 Suppl:44–7. Epub 2012/08/25. S0969-8043(12)00388-0 [pii] 10.1016/j.apradiso.2012.06.023 . 22917941

[5] Hupe O, Ankerhold U. Determination of the dose to persons assisting when X-radiation is used in medicine, dentistry and veterinary medicine. Radiat Prot Dosimetry. 2011;144(1–4):478–81. 10.1093/rpd/ncq351 . 21062800

[6] Flynn M, Gingold E, Goldman L, Krugh K, Leong DL, Mah E, et al An Exposure Indicator for Digital Radiography. College Park, MD, USA: American Association of Physicists in Medicine, 2009 0271–7344.

[7] Gibson DJ, Davidson RA. Exposure creep in computed radiography: a longitudinal study. Acad Radiol. 2012;19(4):458–62. 10.1016/j.acra.2011.12.003 . 22225727

[8] Ma WK, Hogg P, Tootell A, Manning D, Thomas N, Kane T, et al Anthropomorphic chest phantom imaging e The potential for dose creep in computed radiography. Radiography. 2013;19:207–11. 10.1016/j.radi.2013.04.002

[9] Uffmann M, Schaefer-Prokop C. Digital radiography: The balance between image quality and required radiation dose. European Journal of Radiology. 2009;72(2):202–8. 10.1016/j.ejrad.2009.05.060 WOS:000272787100003. 19628349

[10] Alves AF, Miranda JR, Bacchim Neto FA, Duarte SB, Pina DR. Construction of pediatric homogeneous phantoms for optimization of chest and skull radiographs. Eur J Radiol. 2015;84(8):1579–85. 10.1016/j.ejrad.2015.05.015 . 26044295

[11] Pina D, Duarte S, Netto TG, Morceli J. Phantom development for radiographic image optimization of chest, skull and pelvis examination for nonstandart patient. Applied Radiation and Isotopes. 2009;67:61–9. 10.1016/j.apradiso.2008.07.018 18783956

[12] Samei E, Dobbins JT 3rd, Lo JY, Tornai MP. A framework for optimising the radiographic technique in digital X-ray imaging. Radiat Prot Dosimetry. 2005;114(1–3):220–9. Epub 2005/06/04. 114/1-3/220 [pii] 10.1093/rpd/nch562 . 15933112

[13] Precht H, Tingberg A, Waaler D, Outzen CB. New Developed DR Detector Performs Radiographs of Hand, Pelvic and Premature Chest Anatomies at a Lower Radiation Dose and/or a Higher Image Quality. J Digit Imaging. 2013 Epub 2013/11/14. 10.1007/s10278-013-9635-2 . 24221693 PMC3903973

[14] Pavan AL, Alves AF, Duarte SB, Giacomini G, Sardenberg T, Miranda JR, et al Quality and dose optimization in hand computed radiography. Phys Med. 2015;31(8):1065–69. 10.1016/j.ejmp.2015.06.010 . 26148866

[15] Pina DR, Souza RT, Duarte SB, Alvarez M, Miranda JR. Analysis of biological tissues in infant chest for the development of an equivalent radiographic phantom. Med Phys. 2012;39(3):1357–60. Epub 2012/03/03. 10.1118/1.3685588 . 22380369

[16] Chu RYL, Fisher J, Archer BR, Conway BJ, Goodsit MM. AAPM Report No. 31: Standardized Methods for Measuring Diagnostic X-Ray Esposures. New York, USA: American Association of Physicists in Medicine by the American Institute of Physics; 1990.

[17] Goldston RT, Hoskins JD. Geriatrics & gerontology of the dog and cat. Philadelphia: W.B. Saunders Co.; 1995 xvii, 426 p. p.

[18] Pavan ALM, Pina DRd, Giacomini G, Yamashita S, Ribeiro SM, Duarte SB, et al., editors. Quantification of hand tissues to dose optimization procedures in computed radiology. VI Latin American Conference on Biomedical Engineering; 2014; Parana- Argentina.

[19] Jennings RJ. A method for comparing beam-hardening filter materials for diagnostic radiology. Med Phys. 1988;15(4):588–99. Epub 1988/07/01. . 3211052 10.1118/1.596210

[20] White DR. Tissue substitutes in experimental radiation physics. Med Phys. 1978;5(6):467–79. Epub 1978/11/01. . 366367 10.1118/1.594456

[21] Gray JE. Quality Control in Diagnostic Imaging: A Quality Control Cookbook.: Baltimor Md: University Park Press; 1983.

[22] Pina DR, Duarte SB, Morceli J, Ghilardi Netto T. Development of phantom for radiographic image optimization of standard patient in the lateral view of chest and skull examination. Appl Radiat Isot. 2006;64(12):1623–30. Epub 2006/07/21. S0969-8043(06)00210-7 [pii] 10.1016/j.apradiso.2006.05.006 . 16854589

[23] Pina DR, Duarte SB, Ghilardi Netto T, Trad CS, Brochi MA, de Oliveira SC. Optimization of standard patient radiographic images for chest, skull and pelvis exams in conventional x-ray equipment. Phys Med Biol. 2004;49(14):N215–26. Epub 2004/09/11. . 15357201 10.1088/0031-9155/49/14/n02

[24] Ranger NT, Samei E, Dobbins JT 3rd, Ravin CE. Assessment of detective quantum efficiency: intercomparison of a recently introduced international standard with prior methods. Radiology. 2007;243(3):785–95. 10.1148/radiol.2433060485 17517933 PMC2464291

